# Regulation of exosome production and cargo sorting

**DOI:** 10.7150/ijbs.53671

**Published:** 2021-01-01

**Authors:** Hong Wei, Qi Chen, Li Lin, Chunli Sha, Taoqiong Li, Yueqin Liu, Xinming Yin, Yuhao Xu, Lu Chen, Wujiang Gao, Yuefeng Li, Xiaolan Zhu

**Affiliations:** 1 Reproductive Center, The Fourth Affiliated Hospital of Jiangsu University, Zhenjiang, Jiangsu, 212001, China.; 2 Department of Neurology, Affiliated ZhongDa Hospital, School of Medicine, Southeast University, Nanjiang, Jiangsu, 210009, China.; 3 Central Laboratory of the Fourth Affiliated Hospital of Jiangsu University, Zhenjiang, Jiangsu, 212001, China.; 4 Department of Radiology, The Affiliated Hospital of Jiangsu University, Zhenjiang, Jiangsu, 212001, China.; 5 Department of Neurology, The Affiliated Hospital of Jiangsu University, Zhenjiang, Jiangsu, 212001, China.

**Keywords:** exosomes, production, cargo, sorting

## Abstract

Cellular communication can be mediated by the exchange of biological information, mainly in the form of proteins and RNAs. This can occur when extracellular vesicles, such as exosomes, secreted by a donor cell are internalized by an acceptor cell. Exosomes bear specific repertoires of proteins and RNAs, indicating the existence of mechanisms that control the sorting of molecules into them. Knowledge about loadings and processes and mechanisms of cargo sorting of exosomes is essential to shed light on the physiological and pathological functions of these vesicles as well as on clinical applications involving their use and/or analysis. In this review, we will discuss the molecular mechanisms associated with exosome secretion and their specific cargo sorting, with special attention to the sorting of RNAs and proteins, and thus the outcome and the emerging therapeutic opportunities of the communication between the exosome-producer and recipient cells.

## Biogenesis of exosome

### Biogenesis

**Figure 1:**
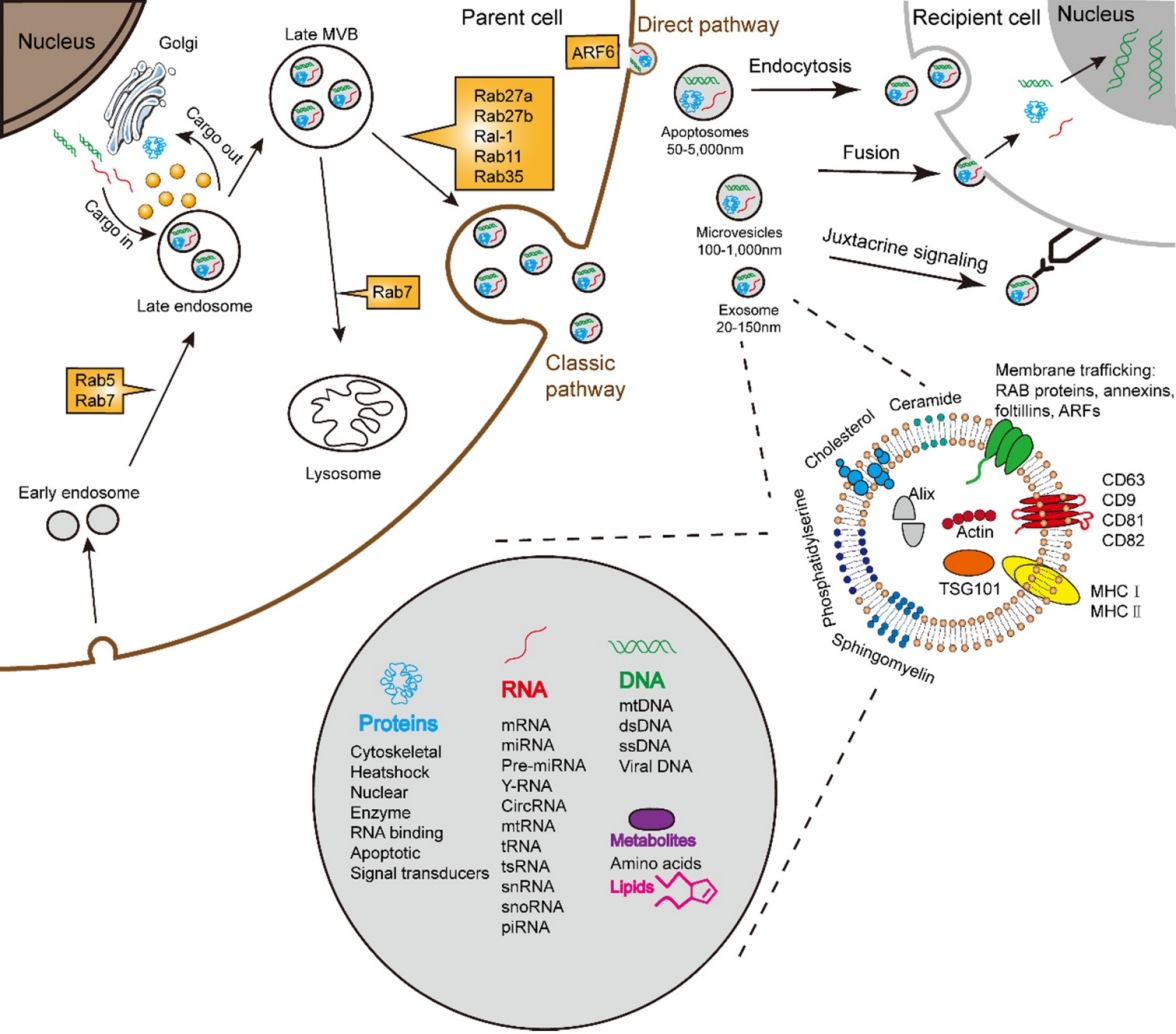
Schematic illustration of exosome biogenesis and composition. Exosomes originate from the inward budding of endosomal multivesicular bodies (MVB). Some of the MVBs formed are transported by associated RAB proteins to fuse with the membrane. MVB can be degraded upon fusion with the lysosome or can release intraluminal vesicles (ILVs) into the extracellular space upon fusion with the plasma membrane. Cells release exosome via the classic pathway and the direct pathway. Subsequently, exosomes can be uptaken by recipient cells via exosomal fusion, endocytosis and juxtacrine signaling to transfer RNA and protein content. Exosomes are surrounded by a phospholipid bilayer and carry various biological species, including proteins, DNA, RNA, lipids and metabolites.

Almost all cells are capable for secreting various types of extracellular vesicles (EVs). In accordance with the origin, functions, and biogenesis, a series of EVs were classified into the following three types: exosomes, microvesicles (MVs)/shedding particles, and apoptosis bodies 1, 2. The “exosome” was coined to describe a group of nanosized (30-150 nm) vesicles that are formed inside endosomes and released into the extracellular environment 3. Exosome biogenesis consists of three processes including the formation of inside endocytic vesicles, the generation of multivesicular bodies (MVBs), and the release of exosomes 4. Apart from this process, MVBs can also be degraded upon fusion with the lysosome. Similar to the host cells, exosomes possess membrane structures composed of a lipid bilayer and contain all known molecular constituents of host cell, including proteins, DNAs, RNAs, lipids and metabolites (**Fig. 1**) 5-7. Essentially, their autocrine, paracrine, and endocrine functions for intracellular or intercellular communication depend on the contents of the exosomes 8.

### Isolation

The isolation of exosome is critical to understanding their mechanisms. Isolation strategies typically used include centrifugation, ultrafiltration, size-exclusion chromatography (SEC), polymer-based precipitation, affinity capture on antibody-coupled magnetic beads, and microfluidics-based technologies 9-11. The gold standard for exosome isolation is widely considered as centrifugation-based techniques. Besides centrifugation, ultrafiltration is one of the most essential methods for the size-based isolation 12, and this method is currently used in combination with ultracentrifugation. SEC enables size-based separation on a single column, with the majority of exosomes eluting before soluble components such as proteins 13. Ultrafiltration and SEC techniques have achieved the high-purity preparations of exosomes 14. Precipitation methods have been used to capture and collect exosomes of a certain size by simple, rapid, low-speed centrifugation 15. Immunoaffinity method is a means via exploit the interactions between proteins and their antibodies and specific interactions between receptors and their ligands 16-18. Recently, microfluidics-based technologies have gradually become important techniques using both the physical and biochemical properties of exosomes 19, 20. The different exosome isolation can affect the analysis and consequently, choosing the appropriate isolation method for different demands should be approached with caution 21.

### Characterization

One of the most vexing problems in exosome biology is how to accurately measure and assess exosome purity 22. The characterization of exosome (such as size, shape, surface charge, and density), is required to determine their action and functions, and hence, the accurate determination of these physicochemical properties are of the utmost importance. There are numerous methods for measuring exosomes, including nanoparticle tracking analyses (NTA), dynamic light scattering (DLS), resistive pulse sensing (RPS), atomic force microscopy (AFM), transmission electron microscopy (TEM), flow cytometry, ELISA, raman spectroscopy, electrochemical detection and microfluidics 23. NTA can provide the size, size distribution, concentration, and phenotype of the exosome 24. The output of DLS is the diameter range of exosome 25. RPS is an essential method which is most useful for investigating cellular function and uptake, and it works when measuring the size distribution and concentration of exosome 26. AFM is a unique alternative to optical and electron diffraction techniques for analyzed exosome 24. TEM is widely used for the visualization of exosome and is used to study the structure, morphology and size of exosome biological components 24. Flow cytometry is used to study exosomal surface proteins 24. ELISA is highly suitable for analyzed exosome from complex bodily fluids. Raman spectroscopy can differentiate exosome as a function of the membrane lipid/protein content along with other various surface modifications 27. Electrochemical detection is widely used for biomolecular analysis 28, 29, and microfluidics has shown great valuable in biological applications 30.

## Formation of exosome

### The classic pathway of exosome formation

The best studied pathway of exosome formation by far is the classic pathway (**Fig. 1**). The classic process of exosome generating involves double invagination of the plasma membrane and the formation of intracellular MVBs containing ILVs 31. The most recognized mechanism for formation of exosome is driven by the endosomal sorting complexes required for transport (ESCRT) complexes 32. The ESCRT is a family of proteins that associate in successive complexes at the membrane of MVBs to sort cargo and the formation of ILVs 33 which are ultimately secreted as exosome through MVB fusion to the plasma membrane and exocytosis 31. Some components of ESCRT could act selectively on MVE and ILVs for exosome secretion 34. Besides ESCRT, a recent study demonstrated the significance of syndecan proteoglycans and their cytoplasmic adaptor syntenin in regulating exosome formation 35. It is reported that exosome biogenesis involves the MVB acidification. Progressive acidification along the endocytic pathway is essential for degradation and recycling of internalized components 36.

A core component of exosome biogenesis involves the ESCRT complexes, while an ESCRT-independent pathway involving ceramides has also been studied 37. The sorting of proteolipid protein (PLP) into ILVs is ESCRT-independent, which is depend on the sphingolipid ceramide 37, 38. Additionally, the tetraspanin family had been proved that regulate ESCRT-independent endosomal sorting 39. The mechanism involved CD63 is required for exosome formation 40. The Rab-related proteins (Rabs) family including several types of small GTPases played a role in the intracellular trafficking of MVEs 41-43. It seems that ESCRT-dependent and ESCRT-independent mechanisms both work in formation of exosome, and their functions may depend on the cargoes and the cell type.

### The direct pathway of exosome formation

Apart from the classic pathway of exosome biogenesis, there is a second route of exosome formation which is much more immediate. The erythroleukemia cell lines and T cells could directly release exosomes from plasma membrane 44-46. These exosomes formed by the direct pathway are indistinguishable from those by the classic pathway.

In sum, exosome formation is certainly complex, which highlights the heterogeneity of the types of exosomes formed by different cells.

## Exosome secretion and uptake

Upon formed, exosomes pinch off from the plasma membrane and prime them for secretion, including autocrine, paracrine and endocrine 39. The mechanisms which underlie exosome maturation and release were therefore important. Exosomes have a different release pathway compared to other exosomes, which can influence their functional properties. Exosome uptake and secretion pathway may intersect; resulting in a mixed population of both endogenously produced and recycled exosomes (**Fig. 1**). The mechanisms and pathways associated with exosome uptake 47, and the specificity of exosome for certain host cell types, add complexity to the biological functions of exosome in intercellular communication.

### Secretion

Recently, it has reported the membrane proteins transfer during direct cell-cell contact 24. Membrane fusion event need to bear the SNAREs, which known as vesicle or v-SNARE and target or t-SNARE 48. The phenomenon of membrane fusion may be regulated by tetraspanin complexes. In the viral-cell fusion, tetraspanins may inhibit cell fusion. In the cell-cell fusion such as mononuclear phagocytes, the inhibition by CD9 and CD81 has also been reported. Membrane fusion related with acidic pH has been a potential mechanism 49, 50.

The eukaryotic and prokaryotic cells both release exosome, which are cup-shaped particles enclosed by a phospholipid bilayer 1. The process of exosome release demands extra steps to sort cargoes into MVBs and ILVs, and additional steps to target MVEs to the membrane and to prime them for secretion 39. The mechanism of exosome release requires their fission from the membrane, and also dependent on the interaction of actin and myosin with a subsequent ATP-dependent contraction 51. Hence, the activation of small GTP-binding proteins leads the exosome bud off from the plasma membranes 52. Exosome release is also induced by stimuli, such as stress, which leads to a rise in intracellular calcium and cytoskeleton remodeling 53. Notably, some exosomes may release via direct outward budding and fission of membrane, analogous to apoptotic blebs.

The process of exosome release may also be subjected to further modulation by Rabs, SNAP and SNARE proteins 54. Rabs are important regulators of exosome transport between different compartments (**Fig. 1**). Rab5 and Rab7 play an essential role in delivering cargo to the early endosomes 55. Rab27a and Rab27b act on the step following MVE transport, which is the docking at membrane to promote fusion 39. The Ral-1 and addition Rabs, such as Rab11 and Rab35, are involved in the direct or potential regulation of MVE secretion 39. The activation of ARF6 allows the vesicles to bud off directly from the membranes 56, 57. The Rab7 and its associated proteins promote the recruitment that targets exosome to lysosomes 58.

### Uptake

Once release, exosomes fuse with the plasma membranes of recipient cells to deliver their contents 59. Alternatively, exosome surface proteins can engage cell surface receptors on recipient cells to induce intracellular signaling 60-62. Several uptake mechanisms have been proposed for exosome 63: endocytosis, membrane fusion, and receptor-ligand mediated interactions (**Fig. 1**) 54, 64. After uptake by different mechanisms, exosomes reach MVEs. The probably the most frequent fate is that exosomes target to lysosomes and ultimate lead to the degradation of exosome cargoes 39. Importantly, this degradative pathway could offer metabolites to the recipient cells 65. Contents delivered by exosomes can also activate various pathological or physiological responses in the recipient cell after internalization. In addition, the recipient cell can also be itself and generate autocrine responses 66. Under certain circumstances, exosomes may escape digestion and release their cargoes into the recipient cells 67.

## Regulation of exosome production and cargo sorting

Upon secreted, exosomes deliver their contents to adjacent or distant cells and play a role of regulating of gene expression and modificating of phenotypes and biology 68, 69. Thus, the contents of exosome are a critical determining factor in their functions.

### Exosome production

#### Membrane protein

The exosomal protein content reflects their origin and varies depending on the types of host cell 70. All exosomes from varies cell types possess a few common sets of proteins. The most commonly proteins are the classes of membrane transport and fusion proteins, and others such as cytoskeletal, metabolic, signaling, and carrier proteins and albumin 71. The exosomal protein most frequently are tetraspanins, which including CD63, CD9, CD81, and CD82, ESCRT-I associated protein (such as Tsg101), lysosome-associated membrane glycoproteins (including LAMP-1 and 2B), MVB-associated protein (Alix-1), heat shock proteins (hsp60, 79, and 90), adhesion molecules (CD45 and CD11b), major histocompatibility molecules (MHC-I and II), Rabs, and membrane-binding proteins (annexins) 72. The ovarian cancers metastasis regulation via MGAT3 mediated glycosylation of tetraspanin CD82 at asparagine 157 73. Moreover, exosomes carry diverse enzymes, including GTPase, metabolic enzymes (including peroxidases, pyruvate and lipid kinases, and enolase-1) 74, with all protein content of exosomes that have been found in the cytosol, plasma membrane or in membranes of endocytic origin 70. These exosomal proteins were not found to consist of proteins of nuclear, mitochondrial, endoplasmic-reticulum or Golgi-apparatus origin 70.

#### Molecular cargo

The plentiful RNA content could result in the greatest source of signaling diversity. Many researches have demonstrated that mRNAs, miRNAs, and other noncoding RNAs contain in exosome 70. They can be absorbed when exosome circulate, which ultimate lead to modify the target gene expression, signal, and overall biological function of receipt cells 75. Exosomal miRNA expression can alter under physiological or pathological conditions. It is known that mRNA was selectively distributed into exosomes, because specific sequences were either preferentially retained or sorted inside the cells 6, 76, 77. Different extracellular miRNA carriers may transport different miRNA sequences 78, 79. There are apparently selective mechanisms that control the specific loading of RNA into exosome, and which eventually resulted in that not all mRNAs species present in a cell end up in exosome. It has been shown that a class of miRNAs sequences are preferentially sorted into exosomes, such as miR-320 and miR-150 75. According to previous study, among small RNAs, the content of miRNA is higher in exosomes than in the host cells 80. These researches all show that host cells hold a sorting mechanism that guides specific miRNAs into exosomes.

#### Lipid composition

Exosomes carry certain lipids, which play an essential role in maintaining the biological activity 37, 81, 82. Exosomes carry cholesterol, sphingolipids, phosphoglycerides, ceramides, and saturated fatty acid chains 48. Importantly, in term of lipid, exosomes gain rigidity and maintain their stability, and facilitating the process of internalization 83. Nevertheless, the lipid content in exosomes does not stand for the host cell 84. Some lipids also have extra biological functions, such as trafficking, recognition, and internalization 85, however, the lipid content of exosome holds limited data.

### Cargo Sorting

**Figure 2:**
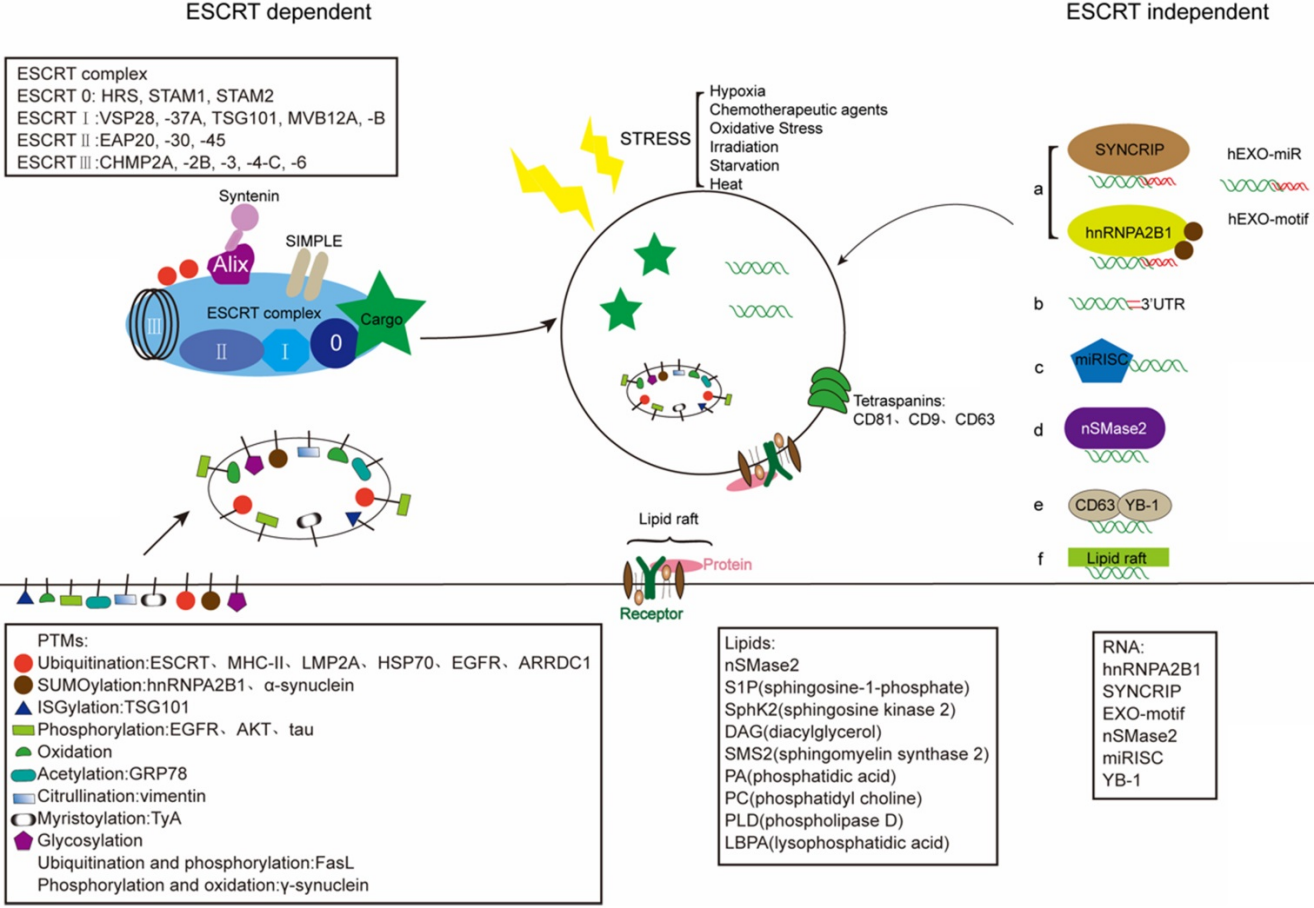
Schematic illustration of exosome sorting. Endosomal sorting complexes required for transport (ESCRT) control the sorting of proteins into exosomes via adding PTMs. Tetraspanins (CD81, CD9, CD63) play a key role in the composition of ESCRT-independent exosomes. And lipid raft-mediated cargo including different lipids and lipid-related enzimes can be loaded into exosomes by the ESCRT-independent pathway. Mature miRNAs are sorted into exosomes via six potential modes: a) miRNA motif and sumoylated hnRNPs-dependent pathway; b) 3'miRNA sequence-dependent pathway; c) the miRISC-related pathway; d) membrane proteins-dependent pathway (e.g. nSMase2); e) other RNA-binding proteins related pathway (e.g. YB-1); f) other potential pathway (e.g. lipid raft). The stress-induced changes in exosomes can influence the composition of exosomes.

Exosomes are enriched with a unique content of proteins, RNAs, and lipids that can vary from the parent cell. The intraluminal content of the exosomal membrane depends on the host cells, and on their cytoplasmatic composition in particular 86. And exosomes allow the transfer of plasma membrane and cytoplasmic components that could reprogram recipient cells. Regarding the sorting mechanism of exosomal cargo, exosomes receive cargoes through the ESCRT-dependent and the independent mechanisms (**Fig. 2**) 87. In addition, the stress, such as hypoxia, also can change the contants of exosome.

#### ESCRT-dependent sorting mechanism

The ESCRT complex has been well acknowledged as a main cargo sorting mechanism. The ubiquitination and ubiquitin-like modifiers represent one of regulatory systems in protein location, stability and function 88. Recent evidences point that ESCRT family involved in sorting ubiquitinated proteins into endosome compartments prior to exosome budding and scission (**Fig. 2**) 89. ESCRT complex performs three functions: it recognizes ubiquitylated cargoes and prevents their degradation; then, it deforms the membrane and sorting cargo in or out; final, it forms ILVs which contain the sorted cargo 90. Proteins are usually loaded into exosome with the help of the ESCRT complex. The ESCRT complex is an important element of the machinery that controls the ubiquitinated proteins into ILVs. The ESCRT complex includes four sub-complexes that successively recognize protein cargoes and sorting into ILVs 88. The ESCRT-I recruited by ESCRT-0 is important to initiat MVB dependent cargoes sorting 91. ESCRT-0 which contains ubiquitin-binding domains (UBDs), could bind tightly to cargo, or even bind with polyubiquitinated cargo 92. Besides ubiquitin-binding motifs, ESCRT-0 could engage cargo through the interactions with the clathrin vesicle machinery 91. The ESCRT-I and ESCRT-II both contain UBDs, while the ESCRT-III subunits hold no UBDs 91. Here, the ESCRT-I component Tsg101 play a functional role in exosome recruitment of Gal3. Tsg101 can specifically recognizes P(S/T)AP sequences via an amino-terminal ubiquitin E2 variant (UEV) domain 93. Nedd4-mediated ubiquitination seems to be an essential process to recruit viral proteins (Gag, LMP2A) 94. It has been shown that while the content of exosomes are not normally hold Nedd4, Nedd4-2 and Itch, Ndfip1 can recruit all these three Nedd4 proteins into exosomes 95. The ability of Nedd4 family recognizes the late (L) domain-containing proteins through the WW domain and the PY motif and sorts into exosomes 96-98. The BAG6 plays a regulatory role on exosome cargoes loading via association with components of the ESCRT complex 99. As described, the motif P(S/T)AP of BAG6 to be crucial for the direct association with Tsg101, an interaction mechanism reminiscent to the ESCRT recruitment during virus assembly 99. Besides binding ubiquitin, the ESCRT complex could be ubiquitinated themselves 88. The ESCRT-dependent mechanism that sorted proteins into ILVs is involved with ubiquitination 91. Before packaging cargo into ILVs, deubiquitination is mandatory 88. ESCRT complex hold the ability of sorting ubiquitinated proteins into ILVs 91. The E3 ubiquitin ligase related with ubiquitin turnover can control the destiny of MVBs. This indicated that deubiquitination is required to sort cargoes into ILVs and exosomes.

However, not all the sorting of proteins depends on ubiquitination. The recent studies show that ubiquitinated proteins can evade deubiquitination. Ubiquitination drives the endocytosis and sorting of MHC II to the luminal vesicles of MVBs for lysosomal degradation 100. It had proved that for MHC II in antigen-loaded DCs, there is an alternative MVB sorting mechanism, that sorting of MHC II to exosomes occurs independently in MHC II ubiquitination 100. In addition to ubiquitination, acetylation can abrogate the sorting of proteins into exosomes. Study has shown that this post-translational modifications (PTMs) serves as a signal for cargo transport into MVBs and demonstrated the ESCRT machinery play a crucial role in this pathway 101. These proteins in exosomes are modified at the position of certain protein residues and are involved in the regulation of MVBs (**Table 1**). In summer, deciphering the sophisticated ESCRT-dependent pathway is necessary to unveil specific functions in the selective import of proteins into exosomes.

#### ESCRT-independent sorting mechanism

##### Protein sorting

PTMs can direct exosomes loading and specific PTMs can control the selective mechanisms of protein cargo sorting and promote some proteins to enrich in exosomes 87. In addition to ubiquitination, SUMOylation, ISGylation, phosphorylation, oxidation, citrullination, glycosylation and myristoylation are availabled for regulating exosome loading and interact between various PTMs 102. Ubiquitination or SUMOylation of proteins worked in sorting into exosomes, while acetylation or ISGylation have an opposed role of driving the modified protein to degradation 103-105. Oxidation and redox processes play a crucial role in exosome cargo sorting. Importantly, oxidation may also affect lipids of the exosome membrane 106. De Jong et al. pointed out that stress, such as hypoxia, could alter the composition of cell-derived exosomes 107. Oxidative stress also influences the RNA composition in exosomes. Of note, recipient cells preconditioned with such exosomes, are protected from oxidative stress, verified the statement from the other side 108. Many studies show that phosphorylation changes the biogenesis of exosome, including ARF6-GTP (ADP ribosylation factor 6) could regulated cargo sorting 109. Glycosylation is a kind of PTMs proteins influencing exosome cargo proteins and membrane. Exosomes, from T-cells, melanoma and colon cancer cells as well as biological fluids, were shared similar glycosylation 102. And as described, adding glycosylating motifs into the specific protein could also mediate its incorporation into exosomes 102, 110. FAT10, containing a carboxyl-terminal GlyGly motif, could modify its target proteins via FAT10ylation to load to exosomes 111. In sum, these PTMs work as a disposal mechanism for cellular harmful components or as a repair mechanism in the pathological or physiological conditions through regulating the release and uptake of exosome. However, the role of these other PTMs remains largely unexplored and this could suggest that a role for these PTMs is unlikely work.

##### Lipid-related sorting

On the other side, growing evidences indicate that the existence of ESCRT-independent mechanisms also play an essential role, including lipid raft, tetraspanin and ceramide-mediated mechanisms. The ESCRT-dependent and independent pathways were not necessarily contradictory, but rather showed the presence of heterogeneous populations of exosome. It has been found that the pathway for intraendosomal transport of proteolipid protein (PLP) may be independent on ESCRT 37. The lipid raft domains of the exosomal membrane can sort some proteins into exosomes 112. Initially, as a mechanism for the selective clearance of clustered membrane receptors and lipids, the lipid rafts involved in exosome sorting was proposed in maturing reticulocytes 113. A recent article pointed out that lipids may be loaded by lipid rafts 114. The lipid raft domains also might constitute the platform for loading into ILVs 115. In addition, in an oligodendrocyte cell line, loading proteolipid protein into exosomes depends on sphingolipid ceramide 37. Moreover, it was reported that a ceramide-dependent mechanism of membrane invagination allowing formation of internal vesicles and cargo loading 37. It has been shown that the nature of the transmembrane and/or luminal domains have a strong influencet on protein loading into exosomes 115. Of note, CD63 induced by ceramide can be blocked by the inhibitor of sphingosine kinase 116. Protein sorting also involved tetraspanin CD63. Cytosolic domains of proteins or lipid domains enriched in the tetraspanin CD9 or CD63 play a role in loading transemembrane proteins to intraluminal vesicles 117, 118. It has been observed that CD63 surrounds the MN envelope and sorts nuclear contents in exosomes 119. It has been demonstrated that CD63 interplays with the RNA binding protein, Y-box protein 1 (YB-1), to sort miR-223 into exosomes, which further supported the mechanism of CD63 in exosome cargo loading 120. For example, this tetraspanin controls the EBV protein LMP1 entry into exosomes 40, and regulates PMEL sorting into ILVs 121, this process independent of the ESCRT complex and ubiquitination 122. CD81-enriched domains have been proved that could work as sorting platforms for exosomal proteins 123 and account for ESCRT-independent cargo sorting and the formation of ILVs population. Tsg101 124, Vps4 125, and ALIX 126 might also be related with the sorting mechanism of proteins or RNAs.

##### RNA and DNA sorting

The exosomal RNAs are not random and the RNAs sorting process is highly selective 127, 128. It has been proved that miRNAs loading in exosomes can be controlled by miRNAs and specific endogenous target sequences 129. First of all, a class of miRNAs are preferentially loaded into exosomes, such as miR-320 and miR-150 75. Based on current research, mechanism of loading for miRNAs summarized as these main pathways and one other potential mechanism 75.

1) The sumoylated heterogeneous nuclear ribonucleoproteins (hnRNPs)-dependent mechanism 75. The miRNAs sequences in exosomes are identified common seed sequences, termed EXO-motifs, that could facilitate binding with RNA-binding proteins, such as hnRNPA2B1 130 and SYNCRIP 131. Some studies prove that short sequence motifs overrepresented in miRNAs (EXO-motifs) that control sorting into exosomes and identify that their directed mutagenesis enables modulation of miRNA cargoes (**Table 2**) 130. The hnRNPA2B1 specifically binds 30 exosomal miRNAs via recogniting GGAG motifs and loads into exosomes 132. The exosomal hnRNPA2B1 is sumoylated, and sumoylation guides the binding of hnRNPA2B1into miRNAs 130. Notably, it is shown that hnRNPA2B1 contains two short RNA recognition motifs (AGG and UAG) in the structure of protein-RNA complex 133. Study identified the GGAG/UGCA motifs for the interaction between the protein hnRNPA2B1 and two miRNAs, miR-198 and miR-601 130. SUMOylation has been shown to control sorting RNA species into exosomes. It has been detected that the SYNCRIP recognized 103 exosomal miRNA sequences, only GGCU is validated 131. SYNCRIP recognizes a specific sequence (GCUG) at the 3′ end of miRNAs for exosome sorting 131. The finding showed that as a component of the miRNA exosomal sorting machinery, the identification of the RNA-interacting protein SYNCRIP, regulated exosome-enriched miRNAs loading 131. Moreover, hnRNPA1 was found to mediate miRNAs like miR-522 packing into exosomes 134. Besides a specific motif (GUUG), it was detected to sort miR-582-5p into exosomes 135. Study found that the GCAG motif present in the miR-1246 controls exosomal miR-1246 enrichment 136.

2) The 3'-end of the miRNA sequence-dependent pathway 137. Exosomal mRNAs cargoes, which enriched in 3'UTR fragments, seem to be essential for specific mRNAs loading into exosomes 138, 139. The CTGCC motif and the miR-1289 specific sequence are shared by other mRNAs enriched in exosomes 139.

3) The miRNA induced silencing complex (miRISC)-related pathway 140. Ago2 has been reported to exert some specific control over the sorting of let-7a, miR-100, and miR-320a into exosomes 141.

4) Membrane proteins related pathway. Membrane proteins like Vps4A and nSMase2 are worked in the miRNA sorting into exosomes. A ceramide-dependent secretory machinery could release some exosomal miRNAs and this process is independent on the ESCRT system 142. Of note, overexpressing nSMase2 could increase the levels of miR-16 and miR-146a in exosomes, while hold no effect on cellular miRNA levels 142. The circRNAs in exosomes are shown to maintain biological activity and can abrogate the growth inhibition induced by miR‐7 in recipient cells 143. Overexpressing Vps4A resulted in increasing levels of exosomal miR-27b-3p and miR-92a-3p in hepatocellular carcinoma cells (HCC), and also caused higher levels of exosome-derived miR-193a-3p, miR-320a, and miR-132-3p 125. On the other side, inhibition of Vps4A resulted in lower levels of exosomal miR-92a and miR-150 144.

5) Other RNA-binding proteins related pathway. The studies proved that specific proteins may control miRNA sorting through recognizing and binding to specific RNAs sequences 131. YB-1 has been demonstrated that it is responsible for the sorting miRNAs and mRNAs into exosomes 120, 145. It has considered that YB-1 could recognize and bind specific mRNA motifs, such as ACCAGCCU, CAGUGAGC and UAAUCCCA 146, 147, and exosomal long ncRNAs (lncRNAs), and control specific lncRNA sorting into exosomes 148. It has proved that YB-1 and NSUN2 as the only proteins interplaying with eRNA-specific motifs, and YB-1 specifically interplays with all three motifs, while NSUN2 only recognizes the motif CAGUGAGC 146, 148. Mutation of YB-1 was identified to impair the sorting miR-144 and miR-223 to exosomes 120, 145, 149. A recent research showed that the cleavage of tRNAs induced by cellular stress causes the production of a class of tRNA fragments containing an oligoguanine motif, which binds YB-1 in a sequence-specific manner 150. And 18 lncRNAs by NSUN2 were associated with exosomes 151. Besides YB-1 and NSUN2, other RNA-binding proteins like MEX3C, Major Vault Protein 4(MVP4), La protein, MTR4 and Anexin-2 work as a key role in exosome cargo sorting 152. MEX3C works as an RNA-binding E3 ubiquitin ligase to mediate mRNA degradation and the MEX3C-Ago2 complex could sort miR-451a into exosomes 153. Collectively, miR-193a is shuttled into exosomes by an MVP-dependent process 154. The La protein is an RNA-binding protein that functions as shuttling miR-122 into exosomes 155. Nuclear exosome adaptors have evolved canonical and non-canonical AIM sequences to target human MTR4 and prove the versatility and specificity with which the MTR4 arch domain can recruit a repertoire of different RNA-binding proteins 73, 156. Anexin-2 might work in sorting RNAs into exosomes, since it binds specific RNAs 157-159 and it is highly enriched in exosomes 160.

6) Other potential pathway. Another ESCRT-independent mechanism may depend on raft-based microdomains for the lateral segregation of cargoes 37. This exosome packaging process depends on a lipid-bilayer binding motif within the specific RNA sequences 161. Ten EXO-motifs (three GCCG, two UGAC, two UCCG, GGAC, GGCG, UGCC) exist in the raft-binding sequence RNA 67-2. Another subset of eleven EXO motifs (three GCCG, two UGAC, two UGCC, GGAC, GGCG, CCCG, GGCC,) is discovered in another raft-binding sequence, RNA 10^161^, ^162^. Of note, overexpressing miR-1289 increases the sorting GalR3 mRNA into exosomes, suggesting that miRNAs could selectively package RNA cargoes into exosomes 87.

As RNA and DNA sorting is still a relatively novel field, it is possible that the current literature only represents studies that have positive findings. Additionally, RNA and DNA sorting is a complicated phenomenon and it is diffcult determine whether the sorting proteins are part of independent sorting mechanisms or whether they involved in a unified larger sorting mechanism.

## Exosome-based strategies for diagnosis and therapy

**Figure 3:**
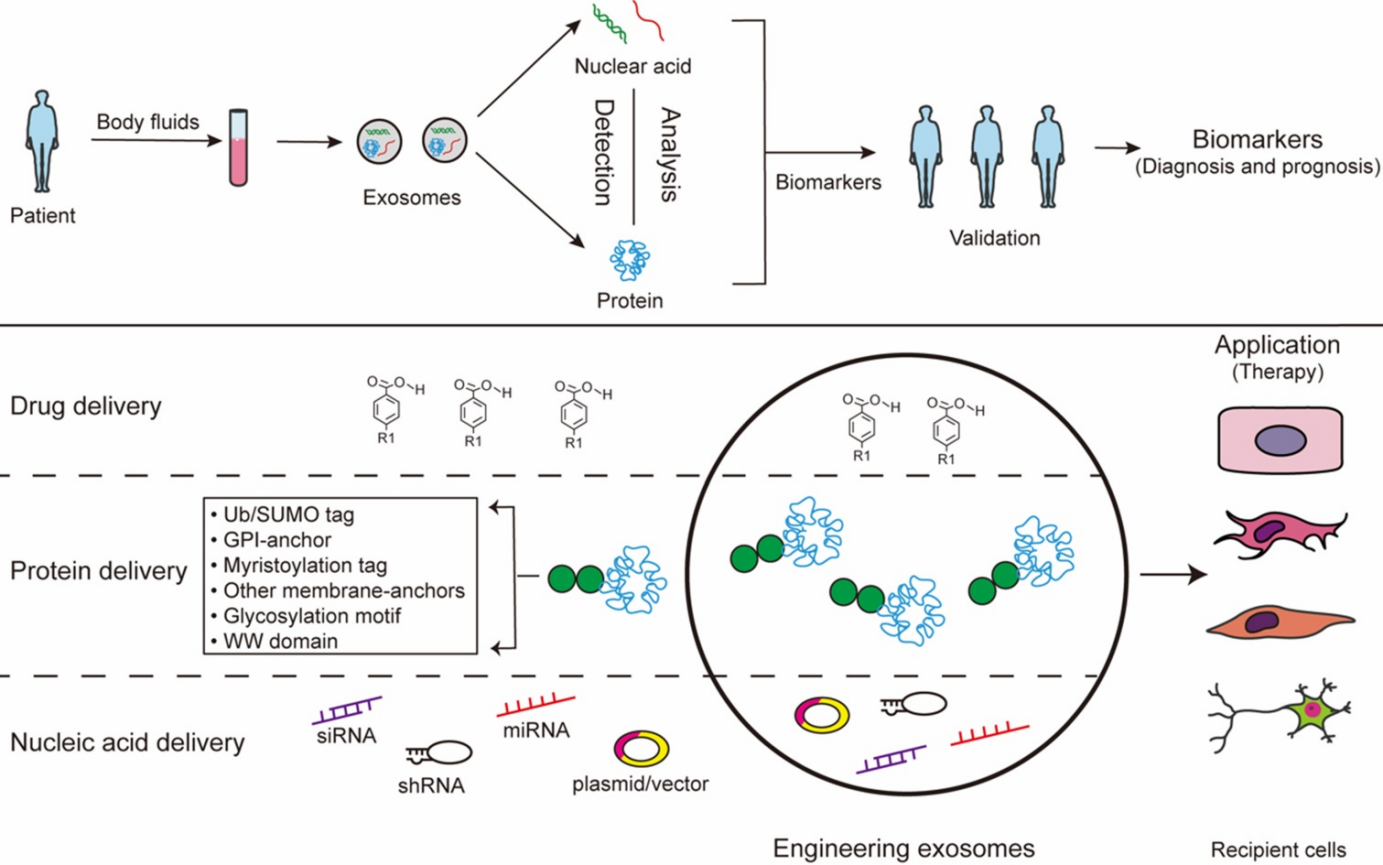
Exosomes for biomedical application. The contants of exosomes obtained from body fluids can be analysed to identify certain condition biomarkers for disease prognosis and diagnosis. Additionally, the exosomes can be modified to contain specific cargoes through bioengineering of exosomes: 1) exosomes can be loaded with chemical drugs for carrier-assisted delivery; 2) exosomes can be targeted by the addition of a specific PTM motif for engineered therapeutic proteins; 3) small interference RNAs can be performed to incorporate inside exosomes therapeutic.

Exosomes exist in all biological fluids and are secreted by all kinds of cells, making them attractive as minimally invasive liquid biopsies with the potential for sampling to follow disease progression 31. Of note, exosomes reflect the metabolic status of the host cells, which mirrors the emerging role of exosome as a fingerprint in the disease diagnosis 163, 164. Studies show that exosomes work as a mechanism similar to that of viruses for coming into cells. Because of viral-like transfection efficiency and inherent biological functions, accumulating evidence reports that exosomes can work as novel delivery platforms for therapy 165. This twin modality of therapeutic and diagnostic is termed theranostics in the emerging field of nanomedicine 165. The long-range targeting and tissue uptake of exosome and their stability in the circulation or in other biological fluids render exosome attractive as a biomarker and a therapeutic vehicle in diseases application (**Fig. 3**).

### Diagnosis

Given their presence in most bodily fluids, exosomes have been wildly known as potential biomarkers for many diseases 166. Cancer cell-derived exosomal miRNAs are different from nonmalignant cells, and such exosomes can be sentinels of disease. Hence, it is theoretically possible to “harvest” these exosomal RNAs, which could provide unique signatures of its host cell, for diagnostic purposes 165. It is observed that serum exosomal miR-223 is a promising biomarker for diagnosing dementia 167. It proved that serum exosomal HOTAIR can be served as a novel prognostic and diagnostic biomarker for glioblastoma multiforme 168. Besides the molecular cargo, exosomal proteins and lipids are also reported as biomarkers. A recent study considered that surviving (an oncoprotein associated with chemoresistance) is overexpressed in patient serum-derived exosomes and works as a diagnostic and prognostic biomarker of prostate cancer (PCa) 169. Tore Skotland et al. showed that lipids in exosomes are promising prostate cancer biomarkers 170. Thus, exosomes have been worked as a potential marker for prediagnosis of diseases by monitoring exosomal contents in the biological fluids. Exosomes can be useful for early diagnosis, targeted therapy, prognosis and clinical monitoring. And exosome diagnosis has a variety of advantages of diagnosis, such as minimally-invasive tissue collection and enrichment in specific exosomal biomarkers. However, the lack of enrichment and time-consuming and expensive identification process still remain a challenge for the clinical application of exosomes.

### Therapy

As the function of exosome cargo has become more widely recognized, exosomes have been proposed as a potential alternative to cell-based therapies. The multifaceted nature of exosome and their compositions underscores the advantage of exosome therapy 171. Exosomes carrying RNAs, miRNAs, or proteins, may be an essential mechanism and the vehicle of their action to reduce inflammation, mediate cellular signaling, and cause tissue repair 172. Compared with exogenous engineering exosomes, the therapeutic effect of endogenous exosomes is limited. Engineering exosomes are promising materials for the next generation of nanomedicine for therapy with non-cytotoxic effects and low immunogenic profiles 173. Engineering exosomes can be packaged with different cargoes, including drugs, recombinant proteins, and nucleic acids like miRNAs, siRNAs. They hold numerous positive attributes that are pivotal in their function as therapy exosome 174. Firstly, exosomes have many features of an ideal drug delivery exosomes. Exosomes containing therapeutic cargo could be generated by sorting exogenous cargo into cells or by directly sorting cargo into exosomes. Drug delivery technology is taking advantage of exosomes as vehicles to overcome biological barriers 175. Exosomes passively loaded with curcumin provide protection against lipopolysaccharide (LPS)-induced microglial activation that causes brain inflammation 176. It is well known that MSC serve as a cost effective exosomes producer for drug delivery. Exosomes derived from MSCs recapitulate the cytoprotective activities and immunomodulatory of their host cells 177. MSC-derived exosomes have been demonstrated in various diseases models, such as respiratory, cardiovascular, neurological, musculoskeletal, hepatic, gastrointestinal, dermatological, and renal disease 178. Secondly, proteins and genetic materials indicate that such biological materials could be packaged into exosomes. Modifications of exosome cargo can confer additional benefits, such as enhanced effects and targeting capability to exosomes. However, the direct sorting of nucleic acids into exosomes may not deliver functionally active cargo into recipient cells efficiently. Thus, RNAs or proteins passively sorted into exosomes by lipofection without cellular cargo loading regulation might be less favorable than RNAs or proteins loaded into naturally occurring or preconditioned cell-derived naive exosomes. The exosome-containing PTM proteins, which called engineered exosomes, enhance their potential as therapeutic tools via selective delivery of therapeutic components into specific tissues. The uptake of exosome containing the C-t domain of PTEN by cancer cells reduces proliferation, migration and metastasis, postulating such a strategy as an anti-tumor treatment 179. The delivery of interfering RNAs is the treatment of different types of cancers, due to the disease-related dysregulation of numerous mRNAs 180, 181. It may be possible to add motifs to RNA in the future so that therapeutic RNA can be better sorted into exosomes to achieve therapeutic effects. Thirdly, liposomes could deliver cargoes shuttle the membrane and provide a barrier against premature transformation and elimination 182. And they are also amenable to *in vivo* and *in vitro* package therapeutic agents, and membrane modifications to enhance tissue-specific homing 182. Fourthly, exosomes are well tolerated as evidenced by their wide distribution in biological fluids 183. Exosomes have an intrinsic ability to home to target tissues, which made the effects of exosomes targeted therapy significantly improved 182 and exosomes have been shown to cross the plasma membrane to deliver their cargo into target cells. Exosomes can be engineered to overexpress specific miRNAs or carrying specific siRNAs that are incorporated into the exosomal cargoes and delivered *in vivo* for the specific targeting of disease. Specially, exosomes have a protective effect on craniocerebral diseases. They have ability to accross the blood-brain barrier, and enhance neural and motor function, which opens a wide variety of possibilities for treatment of neurodegenerative diseases 24. The cargo sorting mechanisms confer specific properties to proteins and RNA, hence, the use of exosome sorting mechanisms represents a tremendous potential tool for the generation of therapeutically personalized and engineering exosomes. Through the study of the exosome sorting mechanisms, nucleic acids and proteins enrichment on engineered exosomes may be used target exosomes to specific cell types and achieve the high-concentration impact targeted therapy. However, the application of exosomes is difficult because of a number of unresolved issues, such as the efficiency of exosome engineering, cost-effectiveness, safety, and biodistribution/pharmacokinetics 184.

## Figures and Tables

**Table 1:** PTM proteins sorting into exosomes. The residues were marked in the brackets

Group	Target Protein(protein residues)
Ubiquitination	Histone H1.2; HLA-G complex;ARRDC1-(Lys, N-terminus, non-lysine residues (Cys, Thr, Ser)); LMP2A-(Lys, N-terminus, non-lysine residues (Cys, Thr, Ser)); PTEN-(Lys, N-terminus, non-lysine residues (Cys, Thr, Ser)); hsp70-(Lys, N-terminus, non-lysine residues (Cys, Thr, Ser)); SIMPLE-(Lys, N-terminus, non-lysine residues (Cys, Thr, Ser)); Aquaporin-1; Annexin A1; Plastin-3 isoform 1; hspX; GroES; GFP; ATG85B-ESAT6; nHer2; MHC-II; Tsg101; Hrs Epsin; Rabex; Eps15; Fas Ligand.
Phosphorylation	CryAB; Tau; EGFR; IRS-1; PDK1; AKT; SRC; ELK1; ERK 1/2; AMPKα1; Acetyl-CoA carboxylase; NCC; Aquaporin 2; GPRC5C; CHMP2B; Annexin A2-(Tyr,Thr,Ser,His); tau-(Tyr,Thr,Ser,His); Fas Ligand-(Tyr,Thr,Ser,His); γ-synuclein-(Tyr,Thr,Ser,His).
SUMOylation	GFP; α-synuclein; hnRNPA2B1
ISGylation	Tsg101
Oxidation	γ-synuclein
Acetylation	GRP78
Citrullination	Vimentin
Myristoylation	TyA-(N-terminal glycine residue)
Glycosylation	LGALS3BP, CD82-(Asn157)
GPI-anchor	CD55
FAT10ylation	FAT10-(carboxyl-terminal GlyGly)

**Table 2:** The motifs of RNAs sorting into exosomes

Group		EXO-motif	mRNA	miRNA	Other noncoding RNA
hnRNPs	hnRNPA2B1	GGAG		miR-654-5p;miR-520e;miR-520b;miR-451;miR-671-5p;miR-1226;miR-422a;miR-193b;miR-198;miR-601;miR-1224-5p;miR-125b-1;miR-125a-3p;miR-483-5p;miR-583;miR-630;miR-513c;miR-513b;miR-513a-5p;miR-188-5p;miR-887;miR-575;miR-765;miR-134;miR-877;miR-1225-5p;miR-638;miR-135a;miR-769-3p;miR-339-3p;miR-939	
		AGG/UAG		miR-17;miR-93	
		GGAG/UGCA		miR-198;miR-601	
		/		miR-503 (Negative)	
					lncARSR
	SYNCRIP	GGCU		miR-6237;miR-409-3p;miR-6337;miR-692;miR-144-5p;miR-369-3p;miR-379-5p;miR-300-3p;miR-410-3p;miR-412-5p;miR-127-5p;miR-381-3p;miR-5108;miR-144-3p;miR-8095;miR-223-3p;miR-6981-5p;miR-122-5p;miR-493-5p;127-3p;miR-3470a;miR-194-2-3p	
		GCUG			
	hnRNPA1			miR-198;miR-522;miR-320;miR-196a	
		GUUG		miR-582-5p	
		GCAG		miR-1246	
3'UTR				miR-486-5p;miR-143-3p;miR-101-3p	
		CTGCC		miR-1289	
miRISC (Ago2)				miR-451;miR--150;miR-142-3p;miR-100;miR-320a;let-7a	
Membrane proteins	Vps4A			miR-27b-3p;miR-92a-3p;miR-92a;miR-150;miR-193a-3p;miR-320a;miR-132-3p	
	nSMase2			miR-210;miR-16;miR-146a	
RNA-binding proteins	YB-1	ACCAGCCU	pre-mRNA		
		CAGUGAGC	SEPT14		
		UAAUCCCA			
				miR-223;miR-133	
				miR-144;miR-233 (Negative)	
		oligoguanine motif			tRNA
	NSUN2	CAGUGAGC	mRNA		
					lncRNA
	MEX3C			miR-451a	
	Major Vault Protein			miR-193a	
	La protein			miR-122	
	MTR4				
	Anexin-2				
Other potential pathway		3 GCCG,2 UGAC,2 UCCG, GGAC, GGCG, UGCC	raft-binding sequence RNA 67-2		
		3 GCCG, 2 UGAC, 2 UGCC, GGAC, GGCG, CCCG, GGCC	raft-binding sequence RNA 10		
